# Isatuximab, carfilzomib, and dexamethasone in patients with relapsed multiple myeloma: updated results from IKEMA, a randomized Phase 3 study

**DOI:** 10.1038/s41408-023-00797-8

**Published:** 2023-05-09

**Authors:** Thomas Martin, Meletios-Athanasios Dimopoulos, Joseph Mikhael, Kwee Yong, Marcelo Capra, Thierry Facon, Roman Hajek, Ivan Špička, Ross Baker, Kihyun Kim, Gracia Martinez, Chang-Ki Min, Ludek Pour, Xavier Leleu, Albert Oriol, Youngil Koh, Kenshi Suzuki, France Casca, Sandrine Macé, Marie-Laure Risse, Philippe Moreau

**Affiliations:** 1 Department of Hematology, University of California at San Francisco, San Francisco, CA USA; 2 The National and Kapodistrian University of Athens, Athens, Greece; 3 Translational Genomics Research Institute, City of Hope Cancer Center, Phoenix, AZ USA; 4 Department of Haematology, University College Hospital, London, UK; 5 Centro Integrado de Hematologia e Oncologia, Hospital Mãe de Deus, Porto Alegre, Brazil; 6 Department of Haematology, Lille University Hospital, Lille, France; 7 Department of Hemato-Oncology, University Hospital Ostrava and Faculty of Medicine, University of Ostrava, Ostrava, Czech Republic; 8 Department of Hematology, 1st Faculty of Medicine, Charles University and General Hospital, Prague, Czech Republic; 9 Perth Blood Institute, Murdoch University, Perth, Australia; 10 Division of Hematology-Oncology, Department of Medicine, Samsung Medical Center, Sungkyunkwan University School of Medicine, Seoul, South Korea; 11 Hospital das Clínicas da Faculdade de Medicina da Universidade de São Paulo, São Paulo, Brazil; 12 Department of Hematology, Seoul St. Mary’s Hospital, The Catholic University of Korea, Seoul, South Korea; 13 Department of Internal Medicine, Hematology and Oncology, University Hospital Brno, Brno, Czech Republic; 14 Service d’Hématologie et Thérapie Cellulaire, CHU and CIC Inserm, 1402 Poitiers Cedex, France; 15 Institut Josep Carreras and Institut Catala d’Oncologia, Hospital Germans Trias I Pujol, Badalona, Spain; 16 Department of Internal Medicine, Seoul National University Hospital, Seoul, South Korea; 17 Myeloma/Amyloidosis Center, Japanese Red Cross Medical Center, Tokyo, Japan; 18 Ividata Life Science, Levallois-Perret, France; 19 Sanofi, R&D, Chilly-Mazarin, France; 20 Sanofi, R&D, Vitry-sur-Seine, France; 21 Department of Hematology, University Hospital Hôtel-Dieu, Nantes, France

**Keywords:** Drug development, Myeloma, Combination drug therapy

## Abstract

Longer-term outcomes with the anti-CD38 antibody isatuximab in combination with carfilzomib-dexamethasone (Isa-Kd) were evaluated in the randomized Phase 3 trial IKEMA (NCT03275285), in a prespecified, follow-up analysis of progression-free survival (PFS, primary study endpoint), final complete response (CR) using Hydrashift Isa immunofixation assay, minimal residual disease (MRD) negativity, and safety. Enrolled patients had relapsed/refractory multiple myeloma (1–3 prior treatment lines). Isa 10 mg/kg was administered intravenously weekly in cycle 1 then biweekly. Efficacy analyses were performed in the intent-to-treat population (Isa-Kd: *n* = 179, Kd: *n* = 123) and safety evaluated in treated patients (Isa-Kd: *n* = 177, Kd: *n* = 122). Consistent with the primary interim analysis, the addition of Isa to Kd prolonged PFS (HR 0.58, 95.4% CI: 0.42–0.79; median PFS 35.7 [95% CI: 25.8–44.0] vs 19.2 [95% CI: 15.8–25.0] months). PFS benefit was observed with Isa-Kd across subgroups, including patients with poor prognosis. The stringent CR/CR rate was 44.1% vs 28.5% (odds-ratio: 2.09, 95% CI: 1.26–3.48), the MRD negativity rate 33.5% vs 15.4% (odds-ratio: 2.78, 95% CI: 1.55–4.99) and the MRD negativity CR rate 26.3% vs 12.2%, with Isa-Kd vs Kd. The safety profile of Isa-Kd was similar to that reported in the prior interim analysis. These findings further support Isa-Kd as a standard-of-care treatment for relapsed multiple myeloma patients.

**Clinical trial information:** ClinicalTrials.gov, NCT03275285.

## Introduction

Substantial advances have been made in the management of patients with relapsed and/or refractory multiple myeloma (RRMM) with the adoption of proteasome inhibitors (PIs) and immunomodulatory drugs (IMiDs) within treatment regimens. This patient population is increasing worldwide due to improved life expectancy and other demographic changes [1–7].

Addition of a monoclonal antibody, such as an anti-CD38 antibody, to a PI (e.g., bortezomib, carfilzomib [K]) or an IMiD (e.g., pomalidomide, lenalidomide) plus low-dose dexamethasone [d], within triplet regimens, has led to further significant improvements in clinical outcomes for patients with RRMM [2, 8–15].

The anti-CD38 monoclonal antibody isatuximab (Isa) exerts anti-myeloma activity through several mechanisms of action including antibody-dependent cell-mediated cytotoxicity, complement-dependent cytotoxicity, and direct induction of apoptotic cell death [9–11]. Isa is approved in multiple countries in combination with pomalidomide and low-dose dexamethasone for patients with RRMM after ≥2 prior therapies, based on the results of the randomized Phase 3 ICARIA-MM study.

Isa was further approved in combination with carfilzomib and low-dose dexamethasone (Isa-Kd) in the United States for RRMM patients after 1–3 prior lines of therapy, in the European Union and other countries for patients with relapsed MM who have received ≥1 prior therapy, and in Japan for patients with RRMM [12–18]. This approval was based on the results of the preplanned interim analysis of the randomized, multi-national, Phase 3 IKEMA study, in which the addition of Isa to Kd demonstrated a statistically significant improvement in progression-free survival (PFS) vs Kd (hazard ratio [HR], 0.53; 99% confidence interval [CI]: 0.32–0.89; one-sided *p* = 0.0007), with clinically meaningful improvement in the rates of very good partial response or better (≥VGPR, 72.6% vs 56.1%) as per independent response committee (IRC), minimal residual disease (MRD) negativity (29.6% vs 13.0%), and complete response (CR, 39.7% vs 27.6%) in the intent-to-treat (ITT) population, along with a manageable safety profile [15].

To evaluate longer term outcomes with Isa-Kd vs Kd in patients with relapsed MM, we conducted a follow-up, prespecified analysis of the IKEMA study population at 159 PFS events. Here, we report updated study results on PFS, CR rate, MRD negativity rate, MRD negativity and CR rate, in the intent-to-treat population, as well as updated safety findings.

## Methods

### Study design and patients

IKEMA was a prospective, randomized, open-label, active-controlled, Phase 3 study (NCT03275285) conducted worldwide, at 69 study centers in 16 countries. Study eligibility and exclusion criteria have been described in detail previously [15]. Briefly, enrolled patients had relapsed and/or refractory MM with 1 to 3 prior treatment lines and measurable evidence of disease (serum M-protein ≥0.5 g/dL and/or urine M-protein ≥200 mg/24 h) [19]. Patients with primary refractory MM, serum free-light chain measurable disease only, or Eastern Cooperative Oncology Group performance status (ECOG PS) > 2 were not eligible. Patients were also excluded if they had received prior carfilzomib therapy, had a contraindication to treatment with dexamethasone, had an estimated glomerular filtration rate (eGFR) <15 mL/min/1.73 m² (by the modification of diet in renal disease formula) or had a left ventricular ejection fraction <40%.

The study protocol was approved by the Institutional Ethics Committee or independent review board at each center. The study was conducted following the Declaration of Helsinki and the International Council for Harmonisation (ICH) Guidelines for Good Clinical Practice. All patients provided written informed consent.

### Treatment

After confirmation of eligibility, patients were randomly assigned (by interactive response technology) in a 3:2 ratio to treatment with Isa-Kd or Kd (control arm). Patients were stratified by the number of prior treatment lines (1 vs >1) and Revised International Staging System stage (I or II vs III vs not classified).

Patients in the Isa-Kd arm received Isa 10 mg/kg intravenously (IV) on days 1, 8, 15, and 22 of the first 28-day cycle, and on days 1 and 15 of subsequent cycles. In both study arms, carfilzomib was administered IV at 20 mg/m^2^ on days 1 and 2 and at 56 mg/m^2^ on days 8, 9, 15, and 16 of cycle 1, followed by 56 mg/m^2^ on days 1, 2, 8, 9, 15, and 16 of subsequent cycles. Dexamethasone 20 mg was administered IV or orally on days 1, 2, 8, 9, 15, 16, 22, and 23. Treatment continued until disease progression, unacceptable toxicity, or patient request to stop study treatment.

### Assessments and endpoints

The blinded IRC continued to review, after the interim analysis, disease assessments for response and progression performed by central laboratory for M-protein quantification, central review of radiological assessments done locally, and local bone marrow aspiration for plasma cell infiltration when applicable. The Hydrashift 2/4 Isa immunofixation electrophoresis (IFE) assay (Sebia, Lisses Evry Cedex, France) [20], which was not available at the time of the interim analysis, was used on banked serum samples to measure endogenous M-protein in samples suspected of Isa interference (at time points before and after the cutoff date of the interim analysis). PFS, the primary study endpoint, was defined as the time from randomization to the first documentation of disease progression or death from any cause, whichever came first. The CR rate was the proportion of patients who achieved a stringent CR (sCR) or CR as best overall response according to the IMWG response criteria [21]. Response and progression based on serum and/or urine M-protein were confirmed based on two consecutive assessments. PFS2 was defined as the time from randomization to disease progression on next-line treatment or death, whichever occurred first. Efficacy assessments were conducted on day 1 of every cycle and at the end of treatment; they were continued monthly for patients who discontinued study treatment without progression.

MRD was evaluated by next-generation sequencing clonoSEQ Assay (Adaptive Biotechnologies, Seattle, WA, USA) with a minimum sensitivity of 10^−4^ [22] in patients with ≥VGPR, when the confirmed best response was reached. The MRD negativity rate was the proportion of patients for whom MRD was negative at a sensitivity of 10^−5^ at any time point after the first dose of study treatment. Adverse events (AEs) and laboratory abnormalities were monitored and graded according to the National Cancer Institute Common Terminology Criteria for Adverse Events (CTCAE) version 4.03. Safety was regularly reviewed by an independent Data Monitoring Committee until the PFS update.

### Statistical analyses

Efficacy analyses were conducted in the ITT population and summarized by randomized treatment. Median PFS (mPFS) and 95% CIs were calculated using the Kaplan-Meier method. HRs were estimated using a Cox proportional-hazard model stratified by stratification factors as entered in the Interactive Response Technology system (ie, number of previous lines of therapy and R-ISS). The odds ratios were also stratified. Treatment-emergent AEs (TEAEs) were evaluated in the safety population (all treated patients). Categorical and ordinal data were summarized using the number and percentage of patients. Continuous data were summarized for each treatment group using the number of available observations, mean, median, standard deviation, minimum, and maximum. SAS 9.4 software (SAS, Cary, NC) was used for all the analyses.

## Results

### Patients

**Fig. 1:**
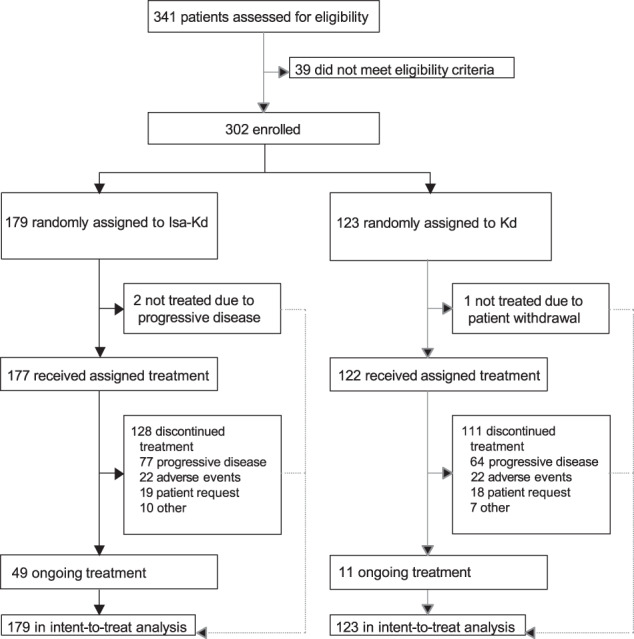
Patient disposition. d dexamethasone, Isa isatuximab, K carfilzomib.

At data cut-off (January 14, 2022), the median follow-up was 44 months; 49 (27.4%) patients in the Isa-Kd arm and 11 (8.9%) in the Kd arm were still on treatment (Fig. 1). The most frequent reason for discontinuation in both arms was progressive disease, although it was less frequent in Isa-Kd vs Kd (43.0% vs 52.0%). Twenty-two patients in each arm discontinued due to an AE (12.3% in Isa-Kd and 17.9% in Kd).

Patient demographic and baseline characteristics were balanced between treatment arms (Table 1). Median age was 65 years in Isa-Kd (range, 37–86) and 63 years in Kd (range, 33–90). ECOG performance status was 0 in 53.1% and 59.3% of patients in Isa-Kd and Kd, respectively. In either treatment arm, at baseline, 24–25% of patients had high-risk cytogenetic status (23.5% in Isa-Kd and 25.2% in Kd) and 42% had 1q21 + (by gain or amplification; 41.9% of patients in Isa-Kd and 42.3% in Kd). The number of prior treatment lines was comparable between study arms, with a median of 2 (range, 1–4) and with 44.1% vs 44.7% of patients with 1 prior treatment line in Isa-Kd vs Kd. A similar incidence of patients had received prior IMiD (76.0% in Isa-Kd and 81.3% in Kd) and/or prior PI therapy (92.7% and 85.4%, respectively). The incidence of lenalidomide-refractory patients was 31.8% in Isa-Kd and 34.1% in Kd.

**Table 1:** Patient demographics and baseline characteristics (ITT population).

	Isa-Kd (*n* = 179)	Kd (*n* = 123)
Age in years, median (range)	65.0 (37–86)	63.0 (33–90)
Age in years, by category, *n* (%)		
<65	88 (49.2)	66 (53.7)
65 – <75	74 (41.3)	47 (38.2)
≥75	17 (9.5)	10 (8.1)
CrCl <60 mL/min/1.73 m² (MDRD)^a^, *n* (%)	43 (26.1)	18 (16.2)
ISS stage at baseline, *n* (%)		
Stage I	89 (49.7)	71 (57.7)
Stage II	63 (35.2)	31 (25.2)
Stage III	26 (14.5)	20 (16.3)
Unknown	1 (0.6)	1 (0.8)
Cytogenetic risk at baseline^b^, *n* (%)		
High	42 (23.5)	31 (25.2)
Standard	114 (63.7)	78 (63.4)
Unknown	23 (12.8)	14 (11.4)
1q21 + , *n* (%)^c^		
Present	75 (41.9)	52 (42.3)
Absent	84 (46.9)	55 (44.7)
Unknown	20 (11.2)	16 (13.0)
Prior lines of therapy, median (range)^d^	2 (1–4)	2 (1–4)
1 line, *n* (%)	79 (44.1)	55 (44.7)
2 lines, *n* (%)	64 (35.8)	36 (29.3)
3 lines, *n* (%)	33 (18.4)	30 (24.4)
Prior proteasome inhibitors, *n* (%)	166 (92.7)	105 (85.4)
Prior IMiDs, *n* (%)	136 (76.0)	100 (81.3)
Patients refractory to, *n* (%)		
IMiD	78 (43.6)	58 (47.2)
Lenalidomide	57 (31.8)	42 (34.1)
PI	56 (31.3)	44 (35.8)
Last regimen	89 (49.7)	73 (59.3)

*CrCl* creatinine clearance, *d* dexamethasone, *eGFR* estimated glomerular filtration rate, *IMiD* immunomodulatory drug, *Isa* isatuximab, *ISS* International Staging System, *ITT* intent-to-treat, *K* carfilzomib, *PI* proteasome inhibitor.

^a^Baseline eGFR by the modification of diet in renal disease (MDRD) formula. Incidence calculated on patients with race reported in case report forms: 165 patients in Isa-Kd arm, 111 patients in Kd arm.

^b^High risk was defined as del(17p), t(4;14), or t(14;16) by fluorescence in situ hybridization. Cytogenetic risk centrally assessed with a 50% cut-off for del(17p) and a 30% cut-off for t(4;14) and t(14;16).

^c^Assessed by central laboratory (cut-off 30%): 1q21+ included gain 1q21 (3 copies) and amplification 1q21 (≥4 copies).

^d^Three patients (1.7%) in Isa-Kd and 2 patients (1.6%) in Kd had >3 prior lines.

### Efficacy

**Fig. 2:**
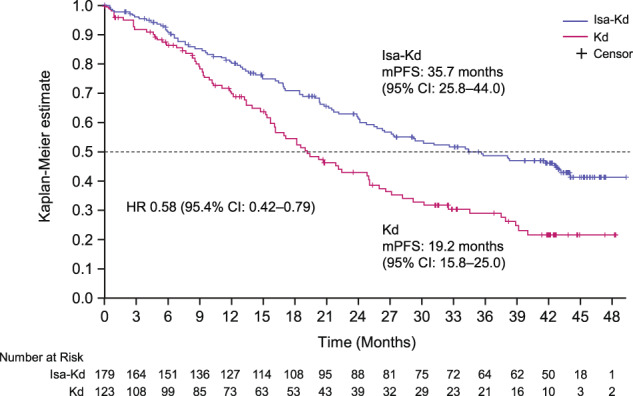
Updated PFS with Isa-Kd vs Kd (ITT population). CI confidence interval, d dexamethasone, HR hazard ratio, Isa isatuximab, ITT intent to treat, K carfilzomib, mPFS median progression-free survival.

Consistent with the interim analysis results [15], the PFS updated analysis, with median follow-up of 44 months, favored Isa-Kd with a HR of 0.58 (95.4% CI: 0.42–0.79), which corresponds to a 42% reduction in the risk of progression or death in the Isa-Kd vs Kd arm. Median PFS (mPFS) reached by Isa-Kd patients was 35.7 (95% CI: 25.8–44.0) months vs 19.2 (95% CI: 15.8–25.0) months in Kd (Fig. 2).

To evaluate the robustness of the primary analysis, PFS sensitivity analyses using different censoring rules, as per IRC and per investigator assessment, were also updated (Supplementary Table S1). All PFS sensitivity analyses showed consistent benefit with Isa-Kd vs Kd, with HRs ranging from 0.57 to 0.64. A further analysis using censoring rules requested and considered by the United States Food and Drug Administration (FDA) as the primary PFS analysis (censoring PFS events occurring >8 weeks from the last valid disease assessment), showed results consistent with the main PFS analysis, with a HR of 0.59 (95.4% CI: 0.42–0.83) and a mPFS of 41.7 months (95% CI: 27.1–not calculable [NC]) with Isa-Kd vs 20.8 months (95% CI: 16.2–28.2) with Kd (Supplementary Table S1, Supplementary Fig. S1).

**Fig. 3:**
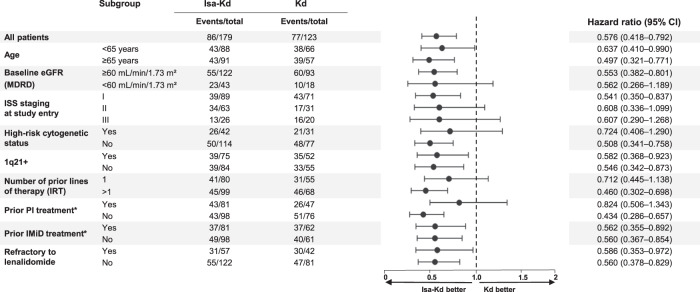
Subgroup analyses of PFS with Isa-Kd vs Kd. CI confidence interval, d dexamethasone, eGFR estimated glomerular filtration rate, IMiD immunomodulatory drug, IRT Interactive Response Technology, Isa isatuximab, ISS international staging system, K carfilzomib, MDRD modification of diet in renal disease equation, PFS progression-free survival, PI proteasome inhibitor. *Prior treatment=last prior anti-myeloma regimen.

As presented in Fig. 3, PFS subgroup analyses, by patient main baseline characteristics and prognostic factors, showed consistent PFS benefit with Isa-Kd vs Kd across all subgroups, including patients with poor prognosis: e.g., elderly patients, patients with renal function impairment, high-risk cytogenetics [del17p, t(4;14) t(14;16)], or 1q21+ status, as well as lenalidomide-refractory patients (PFS subgroup analyses by FDA censoring rules are shown in Supplementary Fig. S2).

**Fig. 4:**
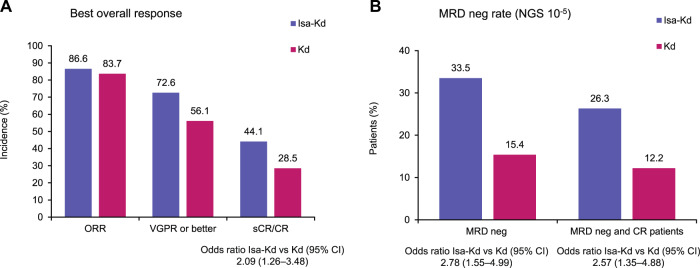
Best overall responses per IRC and MRD negativity rates with Isa-Kd vs Kd (ITT population). **A** Best overall responses, and **B** MRD negativity rates. CR complete response, d dexamethasone, IRC independent response committee, Isa isatuximab, ITT intent to treat, K carfilzomib, MRD minimal residual disease, neg negativity, NGS next-generation sequencing, ORR overall response rate, sCR stringent complete response, VGPR very good partial response.

Although the overall response rates were comparable between treatment arms (86.6% vs 83.7%), deeper responses were seen with Isa-Kd vs Kd in the ITT population: the sCR/CR rate was 44.1% with Isa-Kd vs 28.5% with Kd (odds ratio Isa-Kd vs Kd: 2.09, 95% CI: 1.26–3.48) (Fig. 4A), with additional CRs compared with the interim analysis, 8 in Isa-Kd and 1 in Kd. Half of these 8 additional CRs in the Isa-Kd arm were linked to the availability and use of the Hydrashift 2/4 Isa immunofixation assay (which could detect endogenous IgG M-protein without Isa interference), at time points prior to the interim analysis cut-off (07Feb22).

As shown in Fig. 4B, the addition of Isa to Kd improved the MRD negativity rate to 33.5% vs 15.4% with Kd (odds ratio 2.78, 95% CI: 1.55–4.99 for Isa-Kd vs Kd). The MRD negativity and CR rate with Isa-Kd was 26.3% vs 12.2% with Kd (odds ratio 2.57, 95% CI: 1.35–4.88 for Isa-Kd vs Kd). The MRD negativity rates were consistently higher with Isa-Kd vs Kd across subgroups of patients with poor prognostic characteristics, including older age, renal function impairment, higher ISS stage at study entry, 1q21+ status, more lines of prior therapy, and refractoriness to lenalidomide (Supplementary Fig. S3). Further, exploratory analysis of PFS by MRD status showed that the Kaplan-Meier curves of the MRD-negative patients were largely above those of the MRD-positive patients (Supplementary Fig. S4), with a HR for MRD-negative versus MRD-positive patients of 0.29 (95% CI: 0.17–0.48) in the Isa-Kd arm and 0.19 (95% CI: 0.08–0.47) in the Kd arm (Supplementary Table S2). The medians in MRD-negative patients were not reached in either study arm.

**Fig. 5:**
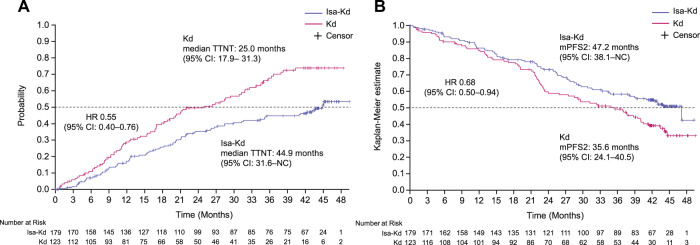
Time to next treatment and PFS2^a^ (ITT population). **A** Time to next treatment and **B** PFS2. CI confidence interval, d dexamethasone, HR hazard ratio, Isa isatuximab, ITT intent to treat, K carfilzomib, mPFS median progression-free survival, NC not calculable, TTNT time to next treatment. ^a^PFS2 was defined as the time from randomization to disease progression on next-line treatment or death, whichever occurred first.

The addition of Isa to Kd also delayed time to the next treatment (TTNT) vs Kd with a HR of 0.55 (95% CI: 0.40–0.76); the median TTNT in the Isa-Kd arm was 44.9 months (95% CI: 31.6–NC) vs 25.0 months (95% CI: 17.9–31.3) in the Kd arm (Fig. 5A). Fewer patients initiated further anti-myeloma therapy in Isa-Kd than in Kd (44.1% vs 64.2%) and among them, 25.3% in Isa-Kd and 60.8% in Kd received anti-CD38 agents: 25.3% vs 54.4%, 1.3% vs 11.4%, and none vs 1.3% of patients in Isa-Kd vs Kd subsequently received daratumumab, Isa, and/or another anti-CD38 agent, respectively (Supplementary Table S3).

Despite the high percentage of patients in Kd who received anti-CD38 in further anti-myeloma therapy, benefit with Isa-Kd over Kd was maintained late through PFS2 with a HR of 0.68 (95% CI: 0.50–0.94) (Fig. 5B). PFS2 was defined as the time from randomization to disease progression on next line of treatment or death, whichever occurred first. Median PFS2 with Isa-Kd was 47.2 (95% CI: 38.1–NC) months vs 35.6 (95% CI: 24.1–40.5) months with Kd. Analysis of overall survival (OS) is planned for 2023, 3 years after the interim PFS analysis that showed significant improvement in PFS. Nonetheless, in the current updated analysis, descriptive OS showed a trend in favor of Isa-Kd vs Kd with HR of 0.78 (95% CI: 0.54–1.12) (Supplementary Table S4).

### Safety

Duration of treatment was longer in the Isa-Kd arm with a median treatment duration of 94.0 (range, 1–215) weeks vs 61.9 (range, 1–208) weeks with Kd (Supplementary Table S5). The median relative dose intensity for Isa was 93.2% (range, 66.7–108.2%) and for carfilzomib it was similar between study arms, despite longer treatment in the Isa-Kd arm: 89.5% (range, 18.2–108.7%) vs 90.8% (range, 41.5–108.6%) in the Kd arm. The median relative dose intensity for dexamethasone was lower in Isa-Kd vs Kd, although it remained above 80% in both arms, at 82.6% (range, 19.3–101.1%) vs 88.1% (range, 23.1–101.6%), respectively. A total of 4405 treatment cycles were administered to Isa-Kd patients and 2181 to Kd patients (Supplementary Table S5).

The safety findings, summarized in Table 2, were consistent with previous findings of the interim analysis [15]; the addition of Isa to Kd did not increase the incidence of TEAEs with fatal outcome during study treatment (5.6% vs 4.9%) or of TEAEs leading to definitive discontinuation of all study treatment (12.4% vs 18.0%).

**Table 2:** Overview of TEAEs and treatment discontinuations (safety population).

*n* (%)	Isa-Kd (*n* = 177)	Kd (*n* = 122)
Any TEAE	175 (98.9)	119 (97.5)
Grade ≥3 TEAEs	148 (83.6)	89 (73.0)
Serious TEAEs	124 (70.1)	73 (59.8)
Any TEAE leading to definitive discontinuation	22 (12.4)	22 (18.0)
Any TEAE leading to premature discontinuation		
Isatuximab	1 (0.6)	N.A.
Carfilzomib	31 (17.5)	1 (0.8)
Dexamethasone	23 (13.0)	7 (5.7)
TEAEs fatal during study treatment^a^	10 (5.6)	6 (4.9)

*d* dexamethasone, *Isa* isatuximab, *K* carfilzomib, *N.A*. not applicable, *TEAE* treatment-emergent adverse event.

^a^Two (1.1%) patients in the Isa-Kd arm and 1 (0.8%) in the Kd arm died of Covid-19 infection.

At interim analysis [15], there was no difference in the incidence of all-causality serious TEAEs, whereas a 10% difference was observed in this updated analysis between the Isa-Kd and Kd arms (70.1% vs 59.8%, respectively). However, this difference was related to the longer treatment exposure in Isa-Kd, since evaluation of the event rate per patient year showed a similar incidence of serious TEAEs in the 2 treatment arms (0.58 in Isa-Kd vs 0.62 in Kd) (Supplementary Table S6). Keeping in consideration duration of treatment exposure, the results also showed that grade ≥3 TEAEs remained more frequent in Isa-Kd vs Kd at the final analysis with an event rate per patient year of 1.08 in Isa-Kd vs 0.97 in Kd, although the difference between the 2 arms decreased (1.26 in Isa-Kd vs 1.05 Kd at the interim analysis). With 2 additional years of follow-up, the incidence of treatment-emergent fatal events remained similar between study arms (5.6% vs 4.9%) and the event rate per patient year remained identical to the one reported at interim analysis (0.03 vs 0.03), despite longer exposure in the Isa-Kd arm and the Covid-19 pandemic (Supplementary Table S6).

As listed in Table 3, the most common, non-hematologic TEAEs were infusion reactions (45.8% vs 3.3%) (all grades 1–2, except for a grade 3 reported as related to carfilzomib), diarrhea (39.5% vs 32.0%), hypertension (37.9% vs 35.2%), upper respiratory tract infection (37.3% vs 27.0%), and fatigue (31.6% vs 20.5%), similar to those reported at interim analysis [15]. Pneumonia occurred in 48 (27.1%) patients in Isa-Kd and 26 (21.3%) patients in Kd (grade ≥3 in 18.6% vs 12.3%). Grade ≥3 fatigue was reported in 5.6% of patients in the Isa-Kd arm and 0.8% in the Kd arm. The incidence of cardiac failure events remained similar in both study arms (8.5% vs 7.4%) [15]. The incidence of second primary malignancies during all study periods (study treatment and post study treatment) was similar between the 2 arms (9.0% vs 7.4%). By type of cancer, the incidence of skin cancer was slightly higher in the Isa-Kd arm (6.2%) than in Kd (3.3%). None led to study treatment discontinuation.

**Table 3:** Most common TEAEs (in ≥20% of patients, safety population) and selected TEAEs.

*n* (%)	Isa-Kd (*n* = 177)		Kd (*n* = 122)	
	All grades	Grade ≥ 3	All grades	Grade ≥ 3
**Most common TEAEs (preferred terms), in** ≥ **20% of patients in the Isa-Kd arm**				
Infusion reaction	81 (45.8)	1 (0.6)	4 (3.3)	0
Diarrhea	70 (39.5)	5 (2.8)	39 (32.0)	3 (2.5)
Hypertension	67 (37.9)	40 (22.6)	43 (35.2)	28 (23.0)
Upper respiratory tract infection	66 (37.3)	6 (3.4)	33 (27.0)	2 (1.6)
Fatigue	56 (31.6)	10 (5.6)	25 (20.5)	1 (0.8)
Dyspnea	54 (30.5)	10 (5.6)	27 (22.1)	1 (0.8)
Pneumonia	48 (27.1)	33 (18.6)	26 (21.3)	15 (12.3)
Back pain	45 (25.4)	3 (1.7)	26 (21.3)	1 (0.8)
Insomnia	45 (25.4)	11 (6.2)	30 (24.6)	3 (2.5)
Bronchitis	43 (24.3)	4 (2.3)	15 (12.3)	1 (0.8)
Arthralgia	39 (22.0)	4 (2.3)	15 (12.3)	2 (1.6)
Cough	39 (22.0)	0	17 (13.9)	0
Asthenia	36 (20.3)	4 (2.3)	20 (16.4)	4 (3.3)
**Selected TEAEs**				
Cardiac failure^a^	15 (8.5)	8 (4.5)	9 (7.4)	5 (4.1)
Second primary malignancy^b,c^	16 (9.0)	8 (4.5)	9 (7.4)	5 (4.1)
Skin cancer	11 (6.2)	2 (1.1)	4 (3.3)	1 (0.8)
Solid tumor, non-skin cancer	7 (4.0)	6 (3.4)	5 (4.1)	3 (2.5)
Hematologic malignancy	0	0	1 (0.8)	1 (0.8)
Not specified	1 (0.6)	1 (0.6)	0	0

*d* dexamethasone, *Isa* isatuximab, *K* carfilzomib, *TEAE* treatment-emergent adverse event.

^a^Groupings using standardized MedDRA query (narrow terms).

^b^Groupings using customized MedDRA query.

^c^Treatment-emergent and post-treatment.

Evaluation of hematologic laboratory abnormalities showed grade 3 anemia in 24.3% vs 21.3% of patients in the Isa-Kd vs Kd arm (with no grade 4 events), grade 3–4 neutropenia in 20.4% vs 7.4% (mainly grade 3, 18.1% vs 6.6%), and grade 3–4 thrombocytopenia in 30% vs 23.8% of patients, respectively (Supplementary Table S7; incidences of anemia, neutropenia, and thrombocytopenia reported as hematologic TEAEs are listed in Supplementary Table S8).

## Discussion

Results of this prespecified PFS updated analysis from IKEMA, at 44-month follow-up, are consistent with the prior interim analysis [15] and demonstrate improvement in PFS with addition of Isa to Kd (HR 0.58 vs Kd, 95.4% CI: 0.42–0.79), with an unprecedented mPFS of nearly 3 years (35.7 [95% CI: 25.8–44.0] months in Isa-Kd vs 19.2 [95% CI: 15.8–25.0] months in Kd), which is the longest PFS reported to date with a PI-based regimen in the relapsed MM setting [23–26]. The extent of such a PFS benefit is in line with the current trend toward more intense therapy earlier in the course of the disease, as PFS generally shortens with each relapse due to the biological characteristics of MM and the development of more aggressive disease over time [27]. The long-term benefit achieved in our study supports the use of the most effective, available drugs and combination therapies as early as possible in relapsed MM, in addition to the newly diagnosed MM setting [28], in order to gain the best outcomes.

In other reports, updated analysis of PFS in the ITT population of the randomized phase 3 study CANDOR, which evaluated a combination of daratumumab with Kd (D-Kd) vs Kd in patients with RRMM, has shown a mPFS of 28.6 months (95% CI: 22.7–not estimable) with D-Kd vs 15.2 months (95% CI: 11.1–19.9) in the Kd arm (HR, 0.59 [95% CI: 0.45–0.78]), at a median follow-up of 27.8 months for D-Kd and 27.0 months for Kd [23]. Although inter-trial evaluations should be interpreted with caution, the HRs for PFS appeared comparable in the updated analyses of IKEMA and CANDOR, whereas mPFS as reported to date was longer in IKEMA. Also, shorter mPFS was observed in the ITT RRMM patient populations of the randomized phase 3 trials CASTOR (D-bortezomib [V]d vs Vd) and OPTIMISMM (pomalidomide-Vd vs Vd), with a mPFS of 16.7 vs 7.1 months (HR, 0.31; 95% CI: 0.25–0.40; *p* < 0.0001) in CASTOR (median follow-up, 40.0 months) and of 11.2 vs 7.1 months (HR, 0.61; 95% CI: 0.49–0.77; *p* < 0·0001) in OPTIMISMM (median follow-up, 15.9 months) [25, 26].

All the PFS sensitivity analyses undertaken in IKEMA using different censoring rules, as per IRC or investigator assessment, strongly favored Isa-Kd over Kd, with HRs ranging from 0.57 to 0.64, adding to the robustness of the study findings. Further analysis showed consistent PFS benefit across all patients subgroups, including patients with poor prognosis, such as older patients (≥65 years of age), patients with renal impairment at baseline (eGFR <60 mL/min/1.73 m^2^), and patients with high-risk cytogenetic status or 1q21+ status by gain or amplification. Median PFS in 1q21+ patients was 25.8 months with Isa-Kd vs 16.2 months with Kd (HR, 0.58; 95% CI: 037–0.92) in favor of Isa-Kd. In addition, also patients who were refractory to lenalidomide derived greater PFS benefit from treatment with Isa-Kd than Kd (HR, 0.59; 95% CI: 0.35–0.97).

In this updated analysis of IKEMA, both the MRD negativity rate (10^−5^) of 33.5% achieved with Isa-Kd vs 15.4% with Kd (odds ratio: 2.78, 95% CI: 1.55–4.99) as well as the MRD negativity and CR rate of 26.3% reached with Isa-Kd vs 12.2% with Kd (odds ratio: 2.57, 95% CI: 1.35–4.88) confirm, after 2 additional years of follow-up, the clinically meaningful difference in favor of Isa-Kd observed at the interim analysis. Such substantial rates of CR and of MRD-negative CR are the highest rates achieved to date with a PI-based regimen in an ITT population of patients with relapsed MM. The finding that the Kaplan-Meier PFS curves of the MRD-negative patients were substantially above those of the MRD positive-patients (HR for MRD-negative versus MRD-positive patients, 0.29 [0.17–0.48] in Isa-Kd and 0.19 [0.08–0.47] in Kd) supports the prognostic relevance of obtaining MRD negativity. It is recognized that MRD negativity could be a surrogate for potential cure in newly diagnosed MM [28] and a recent meta-analysis confirms that also patients with RRMM benefit from reaching MRD negativity [29]. Thus, the MRD negativity rate that can be obtained with a treatment option in RRMM patients is an important criterion to consider, particularly for an optimal management of patients at first relapse. In view of the greater proportion of patients reaching MRD-negative status with Isa-Kd than Kd (33.5% vs 15.4%), Isa-Kd represents a very effective treatment option for patients with relapsed MM, offering the highest likelihood of reaching this status and a better long-term outcome. Numerically, the MRD negativity rate was also higher in the Kd arm of IKEMA than in the control arms of the CANDOR and CASTOR trials [24, 25]. The MRD negativity rate (10^−5^) reported in the CANDOR study, regardless of overall response status, was 22.8% with D-Kd vs 5.8% with Kd (odds ratio: 5.15, *p* < 0.0001) and the best overall MRD-negative CR rate at any time was 13.8% with D-Kd vs 3.2% with Kd (odds ratio: 4.95, *p* < 0.0001) [24]. The MRD negativity rate (10^−5^) in the CASTOR study was 14% with D-Vd vs 2% with Vd in the overall population [25]. Although, as previously mentioned, inter-trial considerations should be made with caution, it is noteworthy that the incidences of patients with prior lenalidomide exposure (40%, 39%, and 36% in the anti-CD38 arms vs 48%, 48%, and 49% in the control arms, respectively) and of patients refractory to lenalidomide (32%, 32%, and 24% in the anti-CD38 arms vs 34%, 36%, and 33% in the control arms, respectively) were similar in the IKEMA, CANDOR, and CASTOR trials [14, 15, 25].

The PFS2 observed in IKEMA favoring Isa-Kd vs Kd (HR: 0.68 [95% CI: 0.50–0.94]) further supports a sustained treatment effect with this Isa triplet combination through subsequent therapies. Consistently, although OS analysis is planned for 3 years after the PFS interim analysis, at a median follow-up of 44 months, results showed a higher probability of survival at 36 and 42 months with Isa-Kd vs Kd: 68.7% vs 62.9% and 66.3% vs 54.5%, respectively.

Median treatment duration with Isa-Kd was longer by ~32 weeks (94.0 vs 61.9 weeks with Kd), with a median relative dose intensity for Isa of 93.2%. The longer treatment in Isa-Kd did not affect the median relative dose intensity for carfilzomib, which was comparable between treatment arms (89.5% in Isa-Kd vs 90.8% in Kd), thus supporting Isa-Kd as a combination regimen that can be given continuously in a feasible manner, without discontinuation of key therapeutic agents. To facilitate treatment administration, a once-weekly schedule of carfilzomib is currently being evaluated in a multi-arm Phase 1b study (NCT02332850) in combination with Isa and dexamethasone in patients with RRMM and in a Phase 2 study (NCT04430894) in combination with Isa-Rd in transplant-eligible patients with newly diagnosed MM. Anti-CD38 therapy with Isa was administered in this trial by IV infusion rather than the subcutaneous (SC) route. However, in this particular regimen, the combination with carfilzomib, which also requires IV administration, would limit the convenience of an Isa SC delivery. Nonetheless, SC administration of Isa is currently being evaluated in combination with pomalidomide-dexamethasone in a Phase 1b study with promising, preliminary results [30].

After 2 additional years of follow-up and notwithstanding the risks associated with the Covid-19 pandemic, the safety profile of treatment with Isa-Kd remains similar to the results previously reported in the interim analysis [15]. The addition of Isa to Kd was not associated with an increase in fatal TEAEs or definitive treatment discontinuations. The higher incidence of any-grade serious TEAEs in Isa-Kd vs Kd was related to the longer treatment exposure in the Isa-Kd arm. Analysis of event rate per year showed that the higher incidence of grade ≥3 TEAEs in Isa-Kd vs Kd was lower in the final analysis (1.08 vs. 0.97) compared with the interim analysis and it did not lead to an increase in the rate of fatal TEAEs (0.03 vs 0.03) or serious TEAEs (0.58 in Isa-Kd vs. 0.62 in Kd). Consistent with the primary analysis [15], the most frequent TEAEs remained infusion reactions, diarrhea, hypertension, and upper respiratory tract infections, mostly of grade 1–2. Hypertension and cardiac failure (all-grade and grade ≥3) were reported with a similar incidence in the 2 arms.

A limitation of our study was the open-label design. However, central laboratory assessments for M-protein, MRD, and baseline cytogenetics (with cytogenetic information available for almost 90% of the patients) contributed to homogenous assessments across study sites and a reduction in the potential bias related to the open-label design. In addition, PFS, best overall response, MRD negativity rate, TTNT, and PFS2 analyses were all performed in the ITT population, and the efficacy analyses conducted per blinded IRC assessment. Utilizing the Hydrashift Isa immunofixation assay, response rates were adjusted after the assessment of residual serum M-protein without Isa interference.

In conclusion, the improvement in PFS observed in this updated analysis of IKEMA, with a mPFS of nearly 3 years, achievement of MRD negativity in a third of Isa-Kd treated patients, reaching MRD negativity and CR in >25% of them, and a manageable safety profile, further support Isa-Kd as a standard of care treatment for patients with relapsed MM.

### Supplementary information

Supplemental Material

## Data Availability

Qualified researchers may request access to patient-level data and related study documents including the clinical study report, study protocol with any amendments, blank case report form, statistical analysis plan, and dataset specifications. Patient-level data will be anonymized and study documents will be redacted to protect the privacy of our trial participants. Further details on Sanofi’s data sharing criteria, eligible studies, and process for requesting access can be found at: https://www.vivli.org/.

## Appendix: Lay summary

**What did this study look at?**

We conducted an updated analysis to evaluate long-term outcomes in the Phase 3 trial IKEMA. In this study, patients with relapsed multiple myeloma received the anti-CD38 monoclonal antibody isatuximab plus carfilzomib and dexamethasone (abbreviated Isa-Kd) or carfilzomib and dexamethasone (Kd) only.

Isatuximab is approved by the regulatory authorities in various countries in combination with carfilzomib-dexamethasone for patients whose disease has returned (with relapsed or treatment-resistant multiple myeloma), based on the first analysis of IKEMA done in 2020.

**What were the methods of the study?**

In this follow-up analysis, we evaluated: progression-free survival (primary endpoint, defined as the time from study start to disease progression or death), number of patients with no evidence of disease in blood and urine (complete response), and number of patients with no detectable residual disease by a highly sensitive test (all measures of efficacy and disease control), as well as safety of treatment with Isa-Kd.

We compared the results with those of patients in the control group who received Kd only.

Isatuximab was given through a vein at 10 mg/kg, once weekly for 4 weeks and then once every 2 weeks. The efficacy analyses were done in all patients: 179 for Isa-Kd and 123 for Kd. Safety was evaluated in patients who received treatment: 177 for Isa-Kd and 122 for Kd. The study also included patients 65 years of age or older and patients with high-risk myeloma characteristics associated with aggressive disease.

**What were the results of this study?**

Our analysis showed that adding isatuximab to Kd significantly prolonged progression-free survival with a 42% reduction in the risk of disease progression or death (hazard ratio: 0.58, 95.4% confidence interval: 0.42–0.79). The median progression-free survival was 35.7 (25.8–44.0) months with Isa-Kd and 19.2 (15.8–25.0) months with Kd.

This benefit was observed with Isa-Kd in all the patient categories, including older patients and patients with difficult-to-treat disease, such as those with high-risk myeloma or no response to prior treatment with lenalidomide.

In addition, 44.1% of patients had a complete response (no evidence of disease in blood and urine) with Isa-Kd compared to 28.5% with Kd. More patients had no detectable residual disease with Isa-Kd (33.5%) than Kd (15.4%).

After 2 additional years of follow-up, the safety observed in this study was similar to that reported in the first analysis of IKEMA.

**What was the main conclusion?**

These long-term results with isatuximab in combination with Kd further support Isa-Kd as a standard-of-care treatment for patients with relapsed multiple myeloma.

**Trial registration and funding**

The IKEMA study is registered at ClinicalTrials.gov: NCT03275285 and was funded by Sanofi.
