# Correction: Isatuximab, carfilzomib, and dexamethasone in patients with relapsed multiple myeloma: updated results from IKEMA, a randomized Phase 3 study

**DOI:** 10.1038/s41408-023-00923-6

**Published:** 2023-09-27

**Authors:** Thomas Martin, Meletios-Athanasios Dimopoulos, Joseph Mikhael, Kwee Yong, Marcelo Capra, Thierry Facon, Roman Hajek, Ivan Špička, Ross Baker, Kihyun Kim, Gracia Martinez, Chang-Ki Min, Ludek Pour, Xavier Leleu, Albert Oriol, Youngil Koh, Kenshi Suzuki, France Casca, Sandrine Macé, Marie-Laure Risse, Philippe Moreau

**Affiliations:** 1 Department of Hematology, University of California at San Francisco, San Francisco, CA USA; 2 The National and Kapodistrian University of Athens, Athens, Greece; 3 Translational Genomics Research Institute, City of Hope Cancer Center, Phoenix, AZ USA; 4 Department of Haematology, University College Hospital, London, UK; 5 Centro Integrado de Hematologia e Oncologia, Hospital Mãe de Deus, Porto Alegre, Brazil; 6 Department of Haematology, Lille University Hospital, Lille, France; 7 Department of Hemato-Oncology, University Hospital Ostrava and Faculty of Medicine, University of Ostrava, Ostrava, Czech Republic; 8 Department of Hematology, 1st Faculty of Medicine, Charles University and General Hospital, Prague, Czech Republic; 9 Perth Blood Institute, Murdoch University, Perth, Australia; 10 Division of Hematology-Oncology, Department of Medicine, Samsung Medical Center, Sungkyunkwan University School of Medicine, Seoul, South Korea; 11 Hospital das Clínicas da Faculdade de Medicina da Universidade de São Paulo, São Paulo, Brazil; 12 Department of Hematology, Seoul St. Mary’s Hospital, The Catholic University of Korea, Seoul, South Korea; 13 Department of Internal Medicine, Hematology and Oncology, University Hospital Brno, Brno, Czech Republic; 14 Service d’Hématologie et Thérapie Cellulaire, CHU and CIC Inserm, 1402 Poitiers Cedex, France; 15 Institut Josep Carreras and Institut Catala d’Oncologia, Hospital Germans Trias I Pujol, Badalona, Spain; 16 Department of Internal Medicine, Seoul National University Hospital, Seoul, South Korea; 17 Myeloma/Amyloidosis Center, Japanese Red Cross Medical Center, Tokyo, Japan; 18 Ividata Life Science, Levallois-Perret, France; 19 Sanofi, R&D, Chilly-Mazarin, France; 20 Sanofi, R&D, Vitry-sur-Seine, France; 21 Department of Hematology, University Hospital Hôtel-Dieu, Nantes, France

**Keywords:** Drug development, Myeloma, Combination drug therapy

Correction to: *Blood Cancer Journal* 10.1038/s41408-023-00797-8, published online 09 May 2023

In the sentence beginning ‘Isa is approved in multiple countries...’ in ‘Introduction’, the term ‘treatment lines’ should have read ‘therapies’. The full text is as follows.

Isa is approved in multiple countries in combination with pomalidomide and low-dose dexamethasone for patients with RRMM after ≥2 prior therapies, based on the results of the randomized Phase 3 ICARIA-MM study.

The original article has been corrected.

