# Supplementary material for: Isatuximab, carfilzomib, and dexamethasone in patients with relapsed multiple myeloma: updated results from IKEMA, a randomized Phase 3 study
Source: Blood Cancer J. 2023 May 9;13(1):72. doi: 10.1038/s41408-023-00797-8 (PMC10166682; doi:10.1038/s41408-023-00797-8)
Supplement: Supplementary file 1 — Supplemental Material [file 41408_2023_797_MOESM1_ESM.pdf]

SUPPLEMENTARY INFORMATION

Isatuximab, carfilzomib, and dexamethasone in patients with relapsed multiple myeloma: Updated results from IKEMA, a randomized Phase 3 study

TABLES and FIGURES

Supplementary Table S1. Additional PFS analyses.

**ITT population**	**Isa-Kd (*n* = 179)**		**Kd (*n* = 123)**		
	***n* (%) events**	**Median, months (95% CI)**	***n* (%) events**	**Median, months (95% CI)**	**HR, 95.4% CI**
Primary analysis: PFS as per IRC censoring for further anti-MM therapy	86 (48.0)	35.65 (25.76–43.96)	77 (62.6)	19.15 (15.77–25.04)	0.58 (0.42–0.79)
Sensitivity PFS analysis: as per IRC without censoring for further anti-MM therapy	99 (55.3)	30.16 (24.12–41.66)	88 (71.5)	18.99 (15.70–23.66)	0.59 (0.44–0.80)
Sensitivity PFS analysis: as per investigator (including symptomatic deterioration)	107 (59.8)	27.33 (21.68–38.11)	89 (72.4)	16.99 (13.96–21.88)	0.62 (0.47–0.84)
Sensitivity PFS analysis: as per investigator (ignoring symptomatic deterioration)	103 (57.5)	29.21 (23.95–39.36)	82 (66.7)	18.20 (15.24–22.44)	0.64 (0.48–0.87)
Sensitivity PFS analysis: as per IRC including initiation of further anti-MM therapy as an event	113 (63.1)	25.49 (20.34–32.59)	102 (82.9)	16.16 (12.72–19.45)	0.59 (0.44–0.77)
Sensitivity PFS analysis: censoring for death PFS events due to Covid 19	83 (46.4)	35.78 (26.28–NC)	76 (61.8)	19.15 (15.77–25.04)	0.57 (0.41–0.78)
PFS primary analysis as per FDA request/sensitivity analysis for other countries: censoring event occurring >8 weeks after last valid assessment[a]	78 (43.6)	41.66 (27.14–NC)	68 (55.3)	20.76 (16.16–28.19)	0.59 (0.42–0.83)

CI confidence interval, *d* dexamethasone, *HR* hazard ratio, *IRC* Independent Response Committee, *Isa* isatuximab, *ITT* intent to treat, *K* carfilzomib, *MM* multiple myeloma, *NC* not calculable, *PFS* progression-free survival.
[a]FDA *post-hoc* request at time of initial filing.

Supplementary Table S2. Analysis of PFS with Isa-Kd vs Kd by MRD status[a].

	MRD-positive			MRD-negative			
	n	**Events, *n* (%)**	**Median, months (95% CI)**	***n***	**Events, *n* (%)**	**Median, months (95% CI)**	**Hazard ratio (95% CI) vs MRD-positive**
Isa-Kd	119	68 (57.1)	21.68 (16.43–27.14)	60	18 (30.0)	NC (NC–NC)	0.29 (0.17–0.48)
Kd	104	72 (69.2)	16.16 (13.44–19.45)	19	5 (26.3)	NC (29.18–NC)	0.19 (0.08–0.47)

CI confidence interval, *d* dexamethasone, *Isa* isatuximab, *K* carfilzomib, *MRD* minimal residual disease, *NC* not calculable, *PFS* progression-free survival.

[a]MRD was evaluated in patients with ≥VGPR, when confirmed best response was reached.

Supplementary Table S3. Further anti-myeloma treatments.

**ITT population**	**Isa-Kd<br>(*n* = 179)**	**Kd<br>(*n* = 123)**
Kaplan-Meier estimates of TTNT in months, median (95% CI)	44.9<br>(31.61–NC)	25.0<br>(17.94–31.31)
Patients with further anti-myeloma treatment, *n* (%)	79 (44.1)	79 (64.2)
Main treatments, *n* (%)		
Alkylating agents	41 (51.9)	42 (53.2)
Proteasome inhibitors	36 (45.6)	26 (32.9)
Bortezomib	22 (27.8)	18 (22.8)
Carfilzomib	6 (7.6)	5 (6.3)
Ixazomib	14 (17.7)	6 (7.6)
Immunomodulators	64 (81.0)	61 (77.2)
Lenalidomide	34 (43.0)	34 (43.0)
Pomalidomide	38 (48.1)	35 (44.3)
Thalidomide	6 (7.6)	6 (7.6)
Anti-CD38 agents	20 (25.3)	48 (60.8)
Daratumumab	20 (25.3)	43 (54.4)
Isatuximab	1 (1.3)	9 (11.4)
Other anti-CD38 agent	0	1 (1.3)
Further transplant	12 (15.2)	14 (17.7)

CI confidence interval, *d* dexamethasone, *Isa* isatuximab, *ITT* intent to treat, *K* carfilzomib, *NC* not calculable, *TTNT* time to next treatment.

Supplementary Table S4. Analysis of OS with Isa-Kd vs Kd.

**ITT population**	**Isa-Kd (*n* = 179)**	**Kd (*n* = 123)**
Deaths, *n* (%)	64 (35.8)	54 (43.9)
Patients censored, *n* (%)	115 (64.2)	69 (56.1)
Median OS (95% CI)	NC (47.18–NC)	NC (38.93–NC)
Stratified HR (95% CI) vs Kd	0.78 (0.54–1.12)	

CI confidence interval, *d* dexamethasone, *HR* hazard ratio, *Isa* isatuximab, *ITT* intent to treat, *K* carfilzomib, *NC* not calculable, *OS* overall survival.

Supplementary Table S5. Treatment exposure.

**Safety population**	**Isa-Kd (*n* = 177)**	**Kd (*n* = 122)**
Median treatment duration, weeks (range)	94.0 (1–215)	61.9 (1–208)
Relative dose intensity (%), median (range)		
Isatuximab	93.2 (66.7–108.2)	-
Carfilzomib	89.5 (18.2–108.7)	90.8 (41.5–108.6)
Dexamethasone	82.6 (19.3–101.1)	88.1 (23.1–101.6)
Total number of cycles	4405	2181
Cycle delayed, *n* (%)	510 (11.6)	235 (10.8)
Between 4 and 7 days	265 (6.0)	133 (6.1)
More than 7 days	245 (5.6)	102 (4.7)

d dexamethasone, *Isa* isatuximab, *K* carfilzomib.

Supplementary Table S6. Safety overview, exposure-adjusted TEAEs – event rate per patient year.

**Safety population**	**Interim analysis**		**Final analysis**	
TEAE overview, %	**Isa-Kd (*n* = 177)**	**Kd (*n* = 122)**	**Isa-Kd (*n* = 177)**	**Kd (*n* = 122)**
Any TEAE	10.94	9.41	9.01	7.78
Grade ≥3 TEAEs	1.26	1.05	1.08	0.97
Grade 5 TEAEs	0.03	0.03	0.03	0.03
Serious TEAEs	0.70	0.72	0.58	0.62
Any TEAE leading to definitive discontinuation	0.07	0.13	0.06	0.12

d dexamethasone; *Isa* isatuximab, *K* carfilzomib, *TEAE* treatment-emergent adverse event.

Supplementary Table S7. Hematologic laboratory abnormalities.

**Safety population** *n* (%)	**Isa-Kd (*n* = 177)**			**Kd (*n* = 122)**		
	**All grades**	**Grade 3**	**Grade 4**	**All grades**	**Grade 3**	**Grade 4**
Anemia	177 (100)	43 (24.3)	0	121 (99.2)	26 (21.3)	0
Neutropenia	100 (56.5)	32 (18.1)	4 (2.3)	55 (45.1)	8 (6.6)	1 (0.8)
Thrombocytopenia	168 (94.9)	32 (18.1)	21 (11.9)	109 (89.3)	19 (15.6)	10 (8.2)

d dexamethasone, *Isa* isatuximab, *K* carfilzomib.

Supplementary Table S8. Hematologic treatment-emergent adverse events[a].

**Safety population**	**Isa-Kd (*n* = 177)**		**Kd (*n* = 122)**	
Preferred term, *n* (%)	**All grades**	**Grade ≥3**	**All grades**	**Grade ≥3**
Anemia	9 (5.1)	8 (4.5)	5 (4.1)	2 (1.6)
Neutropenia	10 (5.6)	9 (5.1)	1 (0.8)	0
Thrombocytopenia	5 (2.8)	4 (2.3)	12 (9.8)	10 (8.2)

d dexamethasone, *Isa* isatuximab, *K* carfilzomib.

[a]Laboratory abnormalities were reported as treatment-emergent adverse events (in addition to laboratory results in the case report forms) only when they led to action taken on study treatment (dose modification or drug withdrawn) and/or when they were serious.

Supplementary Figure S1. PFS with Isa-Kd vs Kd in ITT population, by FDA censoring rules[a].

CI confidence interval, *d* dexamethasone, *FDA* Food and Drug Administration, *HR* hazard ratio, *Isa* isatuximab, *ITT* intent to treat, *K* carfilzomib, *mPFS* median progression-free survival, *NC* not calculable.
[a]PFS primary analysis as per FDA request at the time of initial filing/sensitivity for other countries: censoring event occurring >8 weeks after last valid assessment.

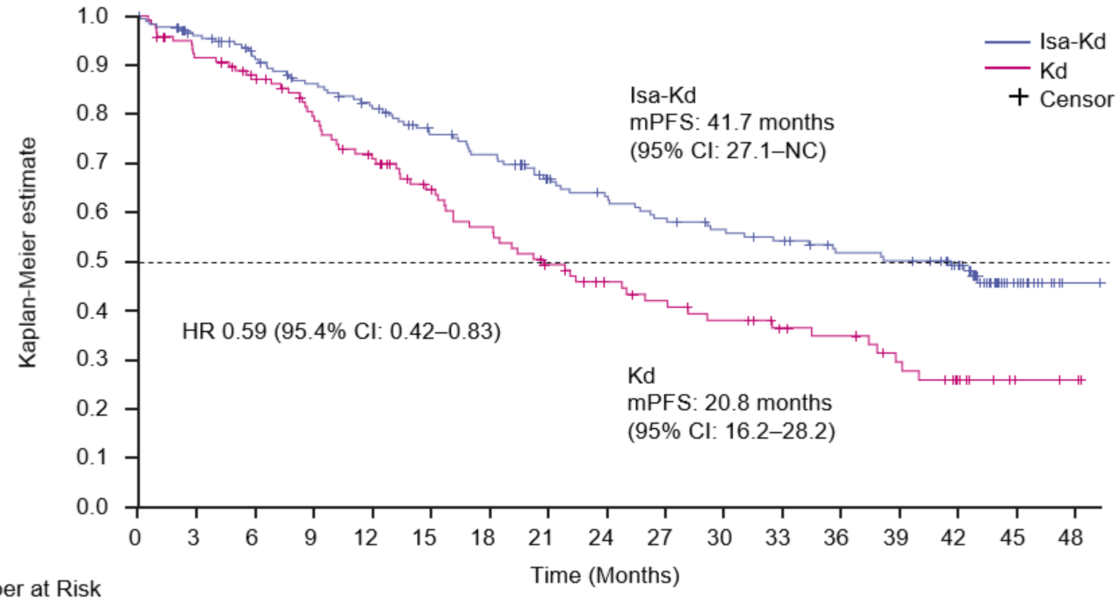

Number at Risk

Isa-Kd	179	163	150	136	127	113	106	92	85	79	74	70	63	61	49	18	1
Kd	123	106	98	83	72	60	52	43	36	32	28	23	21	16	10	3	2

Supplementary Figure S2. Subgroup analyses of PFS with Isa-Kd vs Kd, by FDA censoring rules[a].

CI confidence interval, *d* dexamethasone, *eGFR* estimated glomerular filtration rate, *FDA* Food and Drug Administration, *IMiD* immunomodulatory drug, *IRT*, Interactive Response Technology, *Isa* isatuximab, *ISS* international staging system, *K* carfilzomib, *MDRD* modification of diet in renal disease equation, *PFS* progression-free survival, *PI* proteasome inhibitor.

[a]Analysis as per FDA request at the time of initial filing/sensitivity for other countries: censoring event occurring >8 weeks after last valid assessment. *Analyzed using a non-stratified Cox proportional hazard model with terms for the factor, treatment, and their interaction. At least 1 = high risk; none = standard risk. Prior treatment = last prior anti-myeloma regimen.

Subgroup		Isa-Kd Events/total	Kd Events/total	Hazard ratio (95% CI)*
**All patients**		78/179	68/123	0.594 (0.424–0.832)
**Age**	<65 years	37/88	37/66	0.570 (0.360–0.903)
	≥65 years	41/91	31/57	0.591 (0.369–0.946)
**Baseline eGFR (MDRD)**	≥60 mL/min/1.73 m²	49/122	54/93	0.555 (0.375–0.821)
	<60 mL/min/1.73 m²	22/43	8/18	0.669 (0.296–1.513)
**ISS staging at study entry**	I	35/89	38/71	0.549 (0.345–0.872)
	II	31/63	15/31	0.633 (0.339–1.183)
	III	12/26	15/20	0.609 (0.283–1.307)
**High-risk cytogenetic status**	Yes	25/42	16/31	0.858 (0.457–1.614)
	No	48/114	45/77	0.525 (0.348–0.792)
**1q21+**	Yes	37/75	31/52	0.622 (0.384–1.006)
	No	37/84	29/55	0.586 (0.358–0.957)
**Number of prior lines of therapy (per IRT)**	1	40/80	27/55	0.789 (0.483–1.289)
	>1	38/99	41/68	0.451 (0.288–0.706)
**Prior PI treatment**	Yes	38/81	21/47	0.886 (0.520–1.512)
	No	40/98	47/76	0.448 (0.291–0.688)
**Prior IMiD treatment**	Yes	35/81	34/62	0.582 (0.361–0.939)
	No	43/98	34/61	0.580 (0.368–0.912)

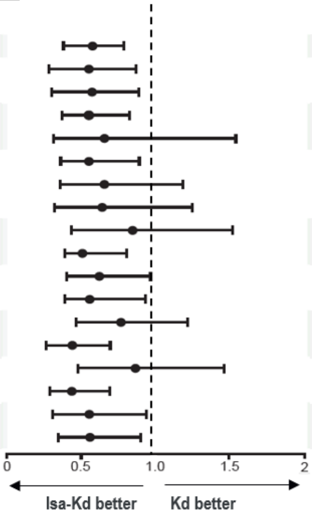

Supplementary Figure S3. Subgroup analyses of MRD negativity with Isa-Kd vs Kd.

d dexamethasone, *eGFR* estimated glomerular filtration rate, *IMiD* immunomodulatory drug, *Isa* isatuximab, *ISS* international staging system, *K* carfilzomib, *MDRD* modification of diet in renal disease equation, *MRD* minimal residual disease, *neg* negativity, *NGS* next-generation sequencing.

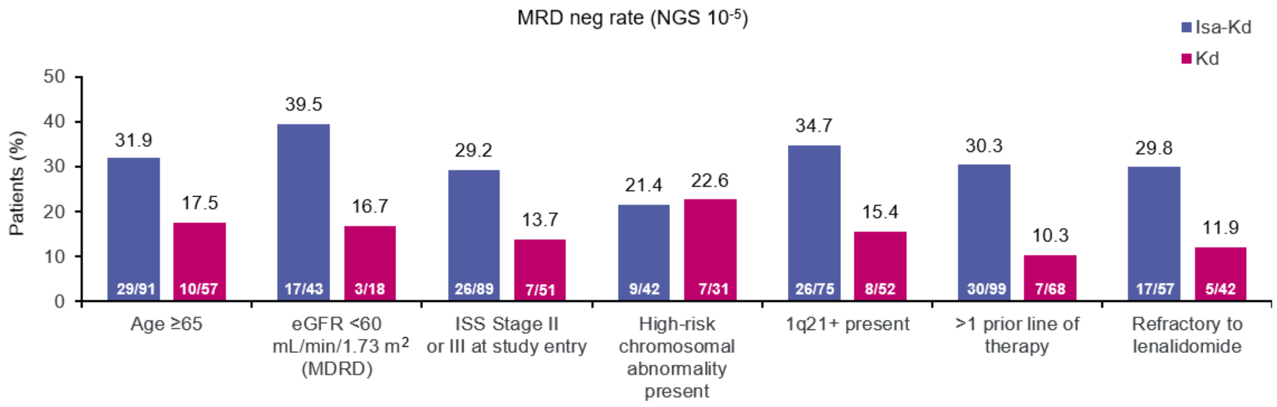

Supplementary Figure S4. Analysis of PFS by MRD status[a].

CI confidence interval, *d* dexamethasone, *HR* hazard ratio, *Isa* isatuximab, *K* carfilzomib, *MRD* minimal residual disease, *neg* negativity, *PFS* progression-free survival.

[a]MRD was evaluated in patients with ≥VGPR, when confirmed best response was reached.

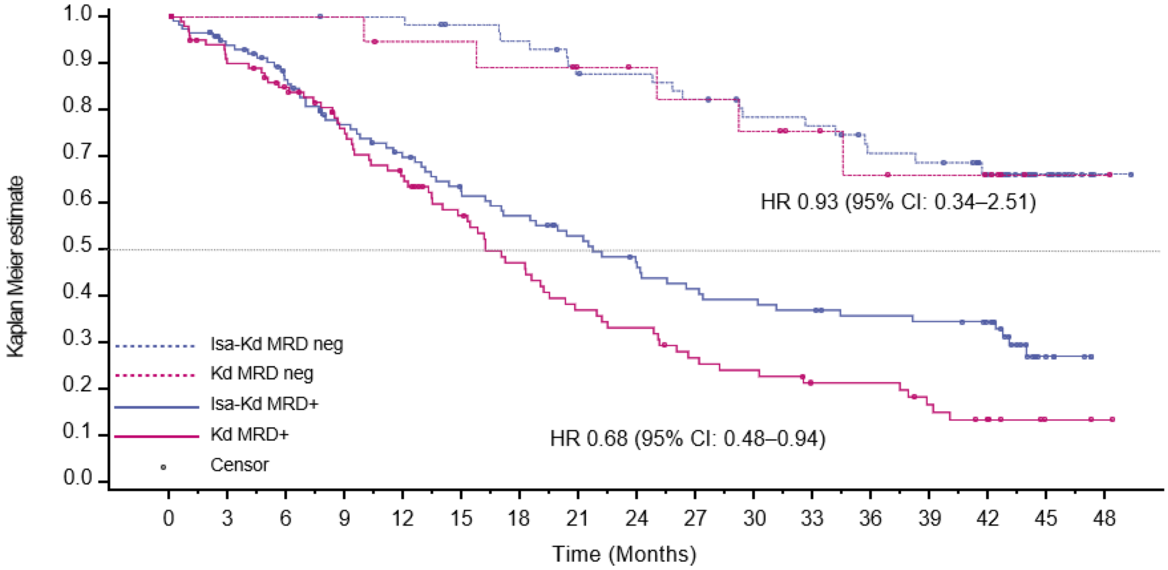

Number at Risk

Isa-Kd MRD neg	60	60	60	59	59	56	54	48	48	45	41	40	35	34	25	13	1
Kd MRD neg	19	19	19	19	17	17	16	14	13	12	11	9	7	6	5	1	1
Isa-Kd MRD+	119	104	91	77	68	58	54	47	40	36	34	32	29	28	25	5	0
Kd MRD+	104	89	80	66	56	46	37	29	26	20	18	14	14	10	5	2	1
